# FLEXBAR—Flexible Barcode and Adapter Processing for Next-Generation Sequencing Platforms

**DOI:** 10.3390/biology1030895

**Published:** 2012-12-14

**Authors:** Matthias Dodt, Johannes T. Roehr, Rina Ahmed, Christoph Dieterich

**Affiliations:** Bioinformatics in Quantitative Biology, Berlin Institute for Medical Systems Biology at the Max Delbrück Centre for Molecular Medicine, Robert-Rössle-Straße 10, 13125 Berlin, Germany; E-Mails: matthias.dodt@googlemail.com (M.D.); johannes.roehr@mdc-berlin.de (J.T.R.); rina.ahmed@mdc-berlin.de (R.A.)

**Keywords:** high-throughput sequencing, demultiplexing, trimming, clipping, quality control

## Abstract

Quantitative and systems biology approaches benefit from the unprecedented depth of next-generation sequencing. A typical experiment yields millions of short reads, which oftentimes carry particular sequence tags. These tags may be: (a) specific to the sequencing platform and library construction method (e.g., adapter sequences); (b) have been introduced by experimental design (e.g., sample barcodes); or (c) constitute some biological signal (e.g., splice leader sequences in nematodes). Our software FLEXBAR enables accurate recognition, sorting and trimming of sequence tags with maximal flexibility, based on exact overlap sequence alignment. The software supports data formats from all current sequencing platforms, including color-space reads. FLEXBAR maintains read pairings and processes separate barcode reads on demand. Our software facilitates the fine-grained adjustment of sequence tag detection parameters and search regions. FLEXBAR is a multi-threaded software and combines speed with precision. Even complex read processing scenarios might be executed with a single command line call. We demonstrate the utility of the software in terms of read mapping applications, library demultiplexing and splice leader detection. FLEXBAR and additional information is available for academic use from the website: http://sourceforge.net/projects/flexbar/.

## 1. Introduction

Next-generation sequencing technologies, such as Illumina Solexa, Applied Biosystems SOLiD or Roche 454, produce millions of short reads by massive parallel sequencing. All of these approaches introduce sequence tags, which are typically ligated to the pool of target sequences. Sequence tags oftentimes overlap with the sequenced region and should be removed prior to downstream read processing. Evidently, adapter sequences may confound any subsequent analysis step. A simple positional read trimming or quality-based read trimming is oftentimes not sufficient to rule out mis-assemblies or low mapping rates. In general, tag sequences could be located anywhere within a given short sequencing read.

Adapter sequences are inherently used by every sequencing platform to initiate sequencing or for other internal processing purposes. As a matter of fact, short reads from any sequencing platform may contain adapters or fragments of adapters. Recent increases in sequencing throughput facilitate pooling of samples (multiplexing) in one sequencing reaction by introducing barcode sequences. Barcode sequences are used to tag a particular origin in a complex mixture of short reads. Several read processing scenarios emerge from using adapter and barcode sequences.

The Flexible Barcode and Adapter Remover (FLEXBAR) software unifies high-processing speed, versatile approaches to basic filtering, quality trimming, demultiplexing, barcode and adapter removal. It supports all current next-generation sequencing platforms, e.g., adapter sequences may be removed in letter-space or color-space. FLEXBAR is not limited in read length and may be well suited for processing third-generation reads. In the following, we will discuss the implementation, program features and compare them to other state-of-the-art software solutions.

## 2. Material and Methods

The rich feature set of FLEXBAR addresses many potential applications in single-end, paired-end and mate-pair sequencing. Typical workflows involve a quality-clipping step, demultiplexing, which potentially includes barcode trimming, followed by a separate adapter trimming step. All of these steps may be executed within the same FLEXBAR program call (see Figure S1). The default parameters of FLEXBAR are optimized to deliver good results (especially Illumina and SOLiD) for a large number of scenarios (see benchmarks). However, customization of settings might improve results for specific applications.

**Figure 1:**
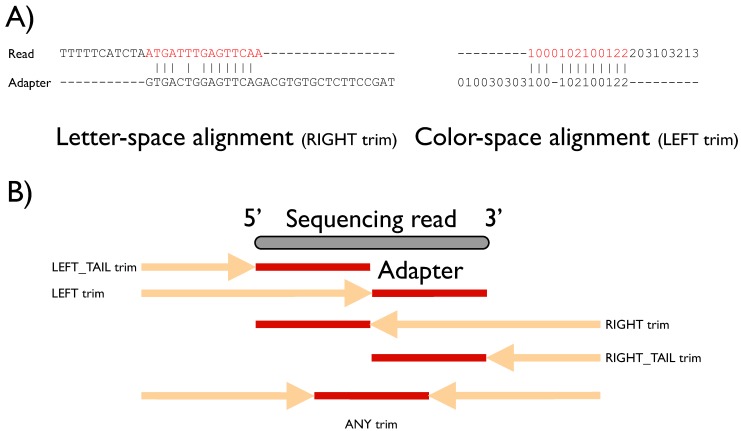
(**A**) Examples of overlap alignments in different sequence encodings. Sequence space and trim mode are denoted under the respective alignments. Red subsequences will be removed from the read sequence. (**B**) Graphical representation of sequence trimming modes. The gray bar depicts the currently processed sequencing read (length n). The best alignment of an adapter sequence (length m; shown in red) might be located anywhere in the demarcated region (arrow + adapter region), which differs according to the selected trim mode (see main text). The name of the trim mode refers to the part of the short read that is removed: in left modes the 5' end is trimmed, right refers to the 3' end, otherwise the shorter end is removed.

FLEXBAR has been implemented in C++ using the Seqan library [1]. Multi-threading has been implemented with the Intel Threading Building Blocks library [2]. FLEXBAR detects target sequences by overlap sequence alignment, based on the Needleman-Wunsch algorithm [3]. An overlap (or semi-global) alignment uses the same recurrence relations as a global alignment but does not penalize gaps at the end of the alignment (Figure 1A and Figure S2). To this end, the first row and column of the dynamic programming matrix are initialized with zeros and the alignment score maximum is searched in the last row and column of the alignment matrix.

FLEXBAR offers maximal flexibility in target sequence recognition by considering base substitutions, insertions and deletions. Moreover, the user is free to choose all alignment scoring parameters, the minimal overlap and a threshold on sequence similarity. Default parameters are preset for these parameters and were chosen to work best for Illumina HiSeq and ABI SOLiD sequencing data. A simple direct matching to expected sequence tags might not be adequate for sequencing platfoms with elevated indel error rates. Furthermore, letter- as well as color-space encoded reads can be processed (Figure 1A). FLEXBAR supports five sequence trimming modes, which cover most sequencing applications: (1) LEFT, (2) LEFT-TAIL, (3) RIGHT, (4) RIGHT-TAIL or (5) ANY(where) trimming (Figure 1B). These modes can be independently combined for adapter and barcode sequence recognition in single or paired-end data. Barcode reads might be even separated from the actual single or paired-end read set (as in Illumina TruSeq^TM^ sequencing).

### 2.1. Trimming Modes

FLEXBAR offers five trimming modes (Figure 1B and Figure S2). Modes should be selected based on the experimental setup. We will explain the different trim-end modes based on a short read of length n and an adapter sequence of length m. All trimming modes are available for barcode (option --barcode-trim-end) and adapter (option --adapter-trim-end) sequence recognition. We assume that m < n holds.

1. **ANY:** The adapter sequence is searched anywhere within the short read. In case of an adapter match, the longer non-matching substring of the read is retained.
2. **LEFT:** The matching adapter sequence is located in a prefix pref[1..k] of the read with k ≤ n. The corresponding short read prefix including the adapter sequence is removed.
3. **RIGHT:** The matching adapter sequence is located in a suffix suff[(n-k)..n] of the short read with k < n. The corresponding short read suffix including the adapter sequence is removed.
4. **LEFT_TAIL:** This is a special case of mode 2. We only consider the first m bases/colors of the short read. The read is trimmed from the 5' end.
5. **RIGHT_TAIL:** This is a special case of mode 3. We only consider the last m bases/colors of the short read. The read is trimmed from the 3' end.

FLEXBAR parameters default to using the ANY mode for barcode sequences and the RIGHT mode for adapter sequences.

### 2.2. Quality Clipping and Read Filtering

There are very good reasons why short reads should undergo a quality control step. Many sequencing platforms provide Phred-scaled quality scores for individual base calls. FLEXBAR provides multiple options to filter reads and target read quality: (1) --max-uncalled sets a threshold on the allowed number of unidentified bases within a given read. No uncalled bases are allowed as per default settings. All reads that exceed this threshold are excluded from barcode and adapter processing. (2) The --pre-trim-left and --pre-trim-right options allow to trim a certain number of bases on the left or right end of short reads. These options are disabled by default. (3) The --pre-trim-phred option trims all read positions from right to left up to the first base/color, that is larger or equal to the given quality score cutoff. This option is disabled per default. (4) The option --post-trim-length specifies the number of bases to which reads are truncated from 3' end after all removal steps have been applied. This option is disabled by default. (5) Finally, the --min-readlength option defines the minimal read length (set to 18 nucleotides per default). All reads that are at least as long as the minimal read length are retained. Reads that are too short are discarded or written to a special output file, since they might not align unambiguously in subsequent read mapping.

### 2.3. Program Usage

A typical use case starts by defining the set of input reads and the read format:

flexbar --target <STRING> --format <STRING> --reads <FILE> [--reads2 <FILE>]
*Example:* flexbar --target processed --format fastq-i1.3 --reads single_end.fastq

Option --target defines the prefix for the output filenames. Option --format selects the read format and, if appropriate, base call quality encoding. If a second read set is specified (option --reads2), paired-end reads are processed and read pairings are maintained in the output. Barcode and/or adapter sequences can be defined by the following options:

--adapters <FASTA FILE> or --adapter-seq <STRING>
--barcodes <FASTA FILE>

The option --adapter-seq is a convenience option if only one adapter sequence needs to be specified. Barcode and adapter sequences may reside within the same read, but could be also disjunct depending on the sequencing setup. For example, barcode reads in the Illumina TruSeq^TM^ system are represented by a second or third read set, which is sequenced independently from the actual single- or paired-end reads. Barcode detection preceds the adapter removal step in FLEXBAR. The user may specify the location of the barcode reads by setting the --barcode-reads option. If this option is not set, barcodes are assumed to reside within the --reads read set. Please note that neither barcode detection nor adapter removal is a mandatory step in FLEXBAR. The available trimming modes are selected by

--adapter-trim-end <ANY|LEFT|LEFT_TAIL|**RIGHT**|RIGHT_TAIL> and
--barcode-trim-end <**ANY**|LEFT|LEFT_TAIL|RIGHT|RIGHT_TAIL>

Default values are shown in bold characters.

The user can adjust how the barcode is detected by refining alignment parameters and whether the barcode is removed or not (option --barcode-keep) from the assigned reads. Adapter removal is effected in a similar manner yet controlled by an entirely different set of parameters: --adapter-* options. This separation of parameters allows for setting more stringent parameters for barcode detection than for adapter sequence detection. For example, this could be motivated by asking for a higher specificity in assigning barcodes and, at the same time, being more sensitive in adapter sequence recognition. Finally, all FLEXBAR processing steps are outlined in Figure S1 and command line parameters plus default settings are output in detail by calling the help page of the program (option --help).

## 3. Results and Discussion

Numerous competing solutions exist for short read processing with an emphasis on adapter trimming and barcode recognition. We conducted a survey of the recent literature and selected the FASTX toolkit [4] as a solution, which is widely accepted by the community. CUTADAPT [5] and BTRIM [6] were selected as recently published solutions. Table 1 is a non-comprehensive list of program features that contrasts FLEXBAR with the other three programs. FLEXBAR is the only software with independent barcode and adapter processing and extensive logging features, e.g., for read alignments. Furthermore, only FLEXBAR preserves read pairs in paired-end or mate-pair data sets, and supports separate barcode reads.

**Table 1:** Comparison of FLEXBAR features with other software.

Feature	FLEXBAR	FASTX	BTRIM	CUTADAPT
Color-space support	Yes	No	No	Yes
Simultaneous barcode & adapter processing	Yes	No	No	No
Preservation of read pairings ^a^	Yes	No	No	No
Alignment logging ^b^	Yes	No	No	No
Separate barcode reads ^c^	Yes	No	No	No

^a^: read pairs are output in sync; ^b^: log files with individual read alignments; ^c^: 1/2 + 1 sequencing format (e.g., TruSeq single or paired-end sequencing plus one extra barcode read).

We evaluated the performance of FLEXBAR by considering four typical use case scenarios. Benchmark 1 focusses on removing adapters from a short read sequencing data set (short read archive (SRA) accession: SRR014966). Briefly, small RNAs from *C. elegans* [7] have been sequenced on the Illumina GA platform, are processed by all four aforementioned programs and mapped back to the genome with the Bowtie aligner (end-to-end alignment). Benchmark 2 deals with a second typical use case: complex processing of a paired-end RNA-seq read set (2 × 100 nt; SRA accession: SRR504324). Benchmark 3 simulates barcode reads with known errors (ground truth) and assesses the ability of FLEXBAR to faithfully recover the correct read assignment. Benchmark 4 is a combination of benchmarks 1 and 3 yet in color space using a recently published data set (SRA accession: SRP008969). Benchmark 4 is motivated by a special application in molecular biology, the detection of trans-splicing events. We use FLEXBAR to recognize splice leader sequences in a nematode data set [8].

### 3.1. Benchmark 1—Adapter Removal from miRNA Short Reads

**Figure 2:**
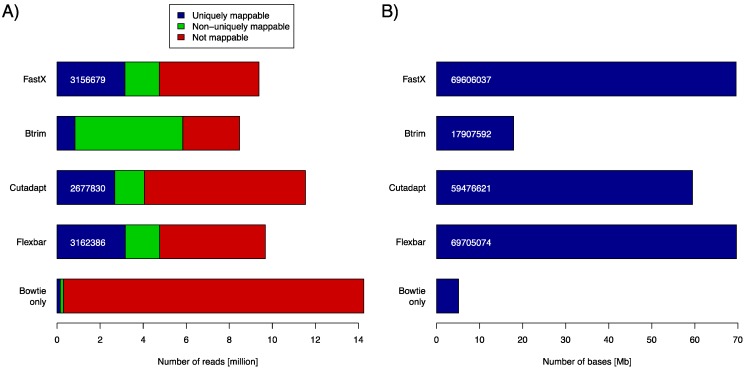
(**A**) Number of returned reads as stratified by subsequent read mapping with Bowtie. Mapping results of untreated reads (Bowtie only) are shown in the bottom most row (control case). The respective adapter removal tools did not return all reads, as some did not pass the respective output filters. (**B**) Number of bases that are contained in all uniquely mappable reads (blue part in A).

Small RNA sequencing is a widely used tool to discover small ncRNAs in general and microRNAs in particular. Typically, small RNA-seq data have read lengths that exceed the size of the target molecules (~21–22 nt for miRNAs). Data set SRR014966 contains short reads of 36 bp. These reads were subject to adapter removal by the four aforementioned software tools. We assessed the tool performance by mapping the retained reads (length ≥ 20 bp) to the *C. elegans* genome using the Bowtie aligner (version 0.12.8). We used the number of uniquely mapped reads and the number of bases contained in uniquely mapped reads as objective performance criteria (Figure 2). Clearly, FASTX and FLEXBAR are the leading solutions in this comparison with FLEXBAR being marginally better than FASTX (69,705,074 *vs*. 69,606,037 uniquely mapped base pairs). BTRIM and CUTADAPT produce significantly less output that can be later uniquely aligned to the genome. Just about 85% of FLEXBAR’s performance (as expressed in uniquely mapped base pairs) is reached by CUTADAPT and only 26% by BTRIM. Exact numbers for Figure 2A and command line options that were used in this evaluation are listed in Table S1 and Table S2, in supplementary material. FLEXBAR is the best performing solution as expressed by the number of unique/total mapped reads.

**Figure 3:**
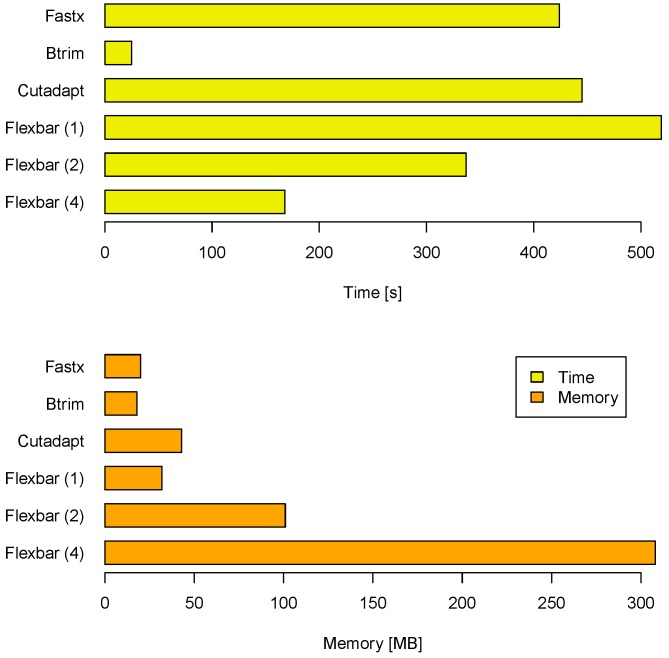
Compute time and memory requirements for FLEXBAR and competitors in benchmark 1. FLEXBAR’s performance is listed for 1, 2 and 4 threads. The evaluation was conducted on a dedicated machine with 2 AMD Opteron 2356 processors, each having 4 cores at 2.3 GHz.

Another aspect of a performance evaluation is the required memory and runtime to arrive at these results (see Figure 3). BTRIM has the lowest resource consumption. FLEXBAR consumes only little more runtime on one core (519 seconds) than CUTADAPT (445 seconds) and FASTX (424 seconds). However, FLEXBAR scales favorable if multiple threads are being used. The observed memory consumption is less of an issue on modern compute systems as indicated by Figure 3. FLEXBAR advantages become even clearer in the second benchmark when intricate processing steps will be performed on the same read set.

### 3.2. Benchmark 2—Trimming and Adapter Removal from Paired-End RNA-seq Data Set

To highlight the conceptional advantages of performing preprocessing steps simultaneously, we present a typical trimming scenario in which several steps are combined. As FASTX is the competitor that showed best results in benchmark 1, we conduct a head-to-head comparison on a paired-end transcriptome dataset (SRA accession: SRR504324). We applied the following series of processing steps on this read set:

1) Filtering reads with uncalled bases
2) Trimming a fixed number of bases on the 5' end of reads
3) Phred score based trimming from 3' end of reads
4) Removal of polyA and polyT tails
5) Retention of reads with a minimal read length

FLEXBAR can perform all these steps within a single program call; whereas several programs of the FASTX toolkit have to be chained together to realize the same processing steps (see Table S3 for command line calls). For example, clipping homopolymer runs of As and Ts requires two program calls instead of one as only one adapter can be specified in FASTX.

More problematic is the fact that FASTX does not handle paired reads natively. All read processing steps have to be performed independently on both read sets yet can be carried out concurrently. We avoid time-consuming file operations by connecting the four required FASTX program calls with UNIX pipes. This is equivalent to allocating eight CPU cores (4 programs × 2 read sets). FASTX and FLEXBAR were allowed to request up to eight CPU cores for this benchmark.

FLEXBAR required only 12 minutes and 26 seconds to complete all processing steps on the whole paired-end read data set. To the contrary, FASTX required 22 minutes and 3 seconds to complete the same challenge with the same compute resources. Most importantly, read pairings are not preserved by the FASTX preprocessing pipeline. Paired reads are not neccesarily kept in sync in the corresponding FASTX output files. This is automatically guaranteed by FLEXBAR and requires no additional software, as is the case for FASTX.

### 3.3. Benchmark 3—Barcode Recognition

Another frequent application of FLEXBAR is demultiplexing in arbitrary barcoding settings. To this end, we have designed an in silico experiment where 8 different 7-mer Illumina TruSeq^TM^ barcodes are attached to the 5' end of “real” reads from benchmark 1 (SRR014966). We have employed the Mason [9] read simulator to generate barcode reads based on an Illumina error model including insertions, deletions and substitutions. We evenly distributed the set of 8 million barcodes over the randomly selected set of 8 million “real” reads. FLEXBAR was then executed on this data set with slightly modified parameters (default parameters are shown in brackets): --max-uncalled 7 (0), --barcode-trim-end LEFT_TAIL (ANY) and --barcode-threshold 2 (1), to allow one mismatch with respect to barcode lengths. The alignment score threshold translates into one tolerated error over the entire 7-mer barcode. We compared FLEXBAR and FASTX (fastx_barcode_splitter.pl tool) on this semi-synthetic read set. Both programs produce exactly the same results: 6 single reads out of 8 million reads were falsely assigned and 86,839 reads could not be assigned to a barcode at all. Consequently, FLEXBAR and FASTX are on a par in our barcode assignment benchmark.

### 3.4. Benchmark 4—C. elegans Color-Space Read Assignment to Splice Leaders

An interesting phenomenon in mRNA processing is trans-splicing. Herein, two mRNA fragments are fused that do not orginate from the same genomic locus. Maxwell *et al*. [8] studied, among others, trans-splicing in the nematode *C. elegans*. They mention the two most relevant splice leader (SL) sequences in their manuscript: “Reads that mapped after stripping and began with GGTTTAATTACCCAAGTTTGAG were counted as being spliced to SL1, whereas those that started with GGTTTTAACCCAGTTACTCAAG were counted as being spliced to SL2”. This is a rather strict way of defining trans-splicing events at the cost of sensitivity. Our third benchmark is an error-tolerant approach to the detection of trans-splicing events. We obtained strand-specific color-space reads of two replicate experiments (SRA accessions: SRR353594 and SRR353599). The library preparation protocol preserves strandedness in the original orientation of transcription (from 5' to 3' end of the transcript). Because of this, splice leader sequences are expected at the 5' end of color-space reads.

**Figure 4:**
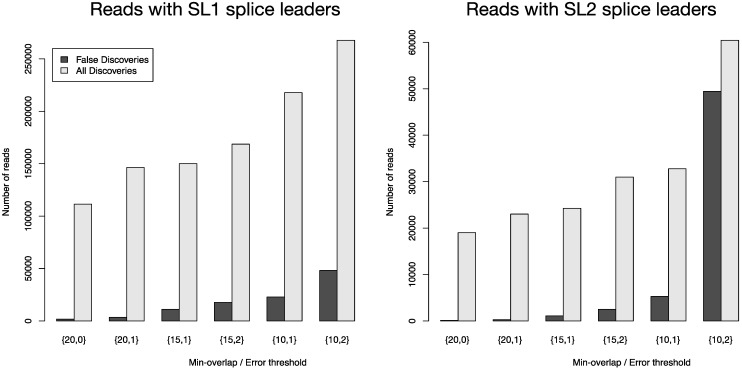
Total number of identified splice leader sequences (light gray bars) and number of estimated false discoveries (black bars) for SL1 and SL2 in data set SRR353594. The following parameters were varied: --barcode-min-overlap {10,15,20}, --barcode-threshold {0,1,2} and --barcode-gap-cost was set to -100. All reads were either assigned to SL1 or SL2 if they passed the alignment criteria (preference is given to SL1 in case of equally scoring alignments). The ratio of all discoveries versus false discoveries is highest for the {20,0} parameter set.

We used the LEFT_TAIL trimming option with different stringency settings to assign trans-splicing events to individual reads and to trim off the identified splice leader sequences for subsequent read mapping. We estimated the false discovery rate of this approach by using the same parameters but with the wrong trimming end (RIGHT_TAIL trimming). Figure 4 depicts the number of all reads that were positively screened for splice leaders versus the number of false discoveries. FLEXBAR offers a flexible way of splice leader sequence recognition. The proportion of false discoveries can be fine-tuned with program parameters. The ratio of all discoveries versus false discoveries is highest for the {20,0} parameter set. Command line calls are listed in Table S4. For read count details see Table S5.

## 4. Conclusions

We have introduced FLEXBAR as a versatile solution to three critical steps in any next-generation read processing pipeline: basic and quality-based clipping, barcode recognition and adapter sequence trimming. FLEXBAR covers a much larger range of sequencing platforms and scenarios, formats and features than other tested solutions, and provides detailed output statistics. It performed slightly better than FASTX, which was the best of all considered competitors in removing adapters from an Illumina short read data set, as measured by the number of uniquely mappable reads and bases. While it consumed only little more runtime on one processor core than FASTX, FLEXBAR scales favorable when using multiple threads. As pinpointed by a paired-end RNA-seq example application, FLEXBAR handles four processing steps in one program call and requires almost 50% less runtime than FASTX. Read pairings are, as a matter of course, preserved in all output files. Furthermore, we demonstrated how faithfully our software recognizes barcodes and avoids false assignments. Finally, we could show that FLEXBAR is also useful for unconventional applications, such as identifying trans-splicing events in a color-space transcriptome data set from *C. elegans*.

## Supplementary Files

Supplementary File 1 Supplementary Material (PDF, 147 KB)
