# Supplementary material for: FLEXBAR—Flexible Barcode and Adapter Processing for Next-Generation Sequencing Platforms
Source: Biology (Basel). 2012 Dec 14;1(3):895–905. doi: 10.3390/biology1030895 (PMC4009805; doi:10.3390/biology1030895)
Supplement: Supplementary File 1 — Supplementary Material (PDF, 147 KB) [file biology-01-00895-s001.pdf]

Supplementary Material

The source code of FLEXBAR and binaries for Linux, Windows and Mac operating systems are available on the website: http://sourceforge.net/projects/flexbar/.

Figure S1. FLEXBAR's internal workflow. Sequencing reads, barcode and adapter sequences are taken as input. FLEXBAR carries out up to 7 processing steps on the corresponding sequence sets. These can be broken up into four blocks: basic clipping and quality filtering, barcode recognition and processing, adapter recognition and processing and output filters.

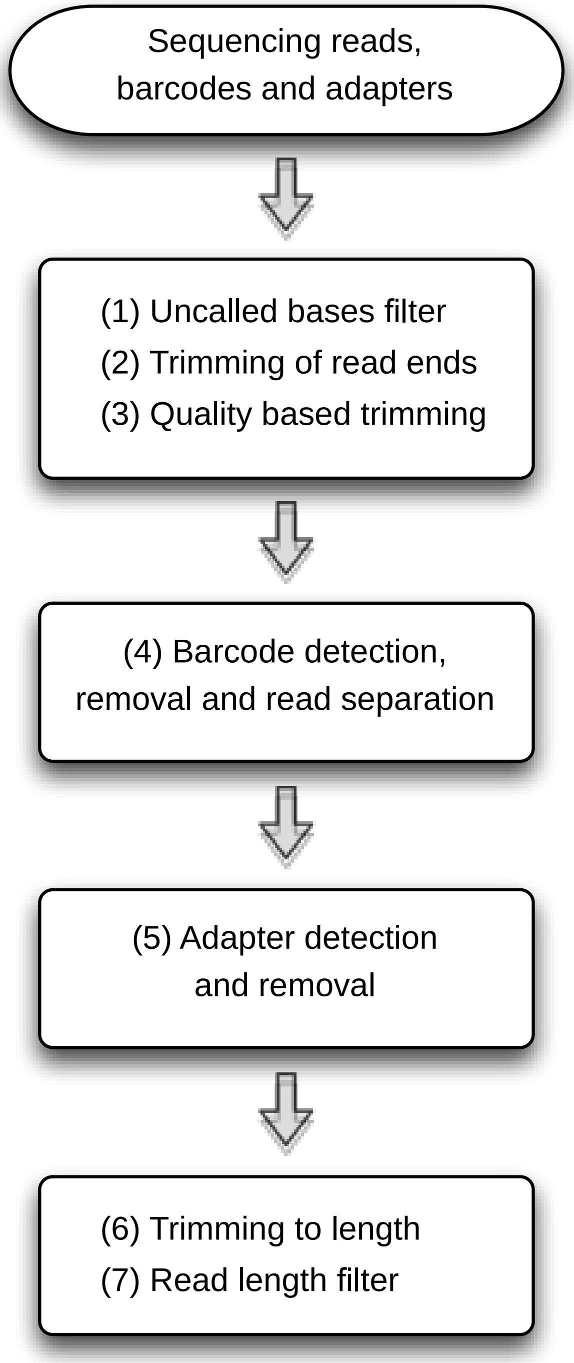

Figure S2. Sequence tag recognition on the dynamic programming matrix. (**A**) Example dynamic programming matrix for trimming mode “ANY” and corresponding overlap alignment. Gaps at either end of any sequence are not penalized: The top-scoring alignment may start at any position in the read (first blue row) or at any position in the sequence tag (first blue column). The maximal score of an alignment could be located at any position in the read (score maximum in last orange row) or sequence tag (score maximum in last orange column). The arrows in the dynamic programming matrix represent the depicted alignment. Case (**B**) and (**C**) represent the left-end and right-end trimming modes, respectively. For case (**B**), an alignment could start anywhere in the blue area, but might only end in the last orange row. For case (**C**), an alignment could end in the orange area, but has to start in the first blue row. LEFT_TAIL and RIGHT_TAIL modes truncate the read length to the sequence tag length.

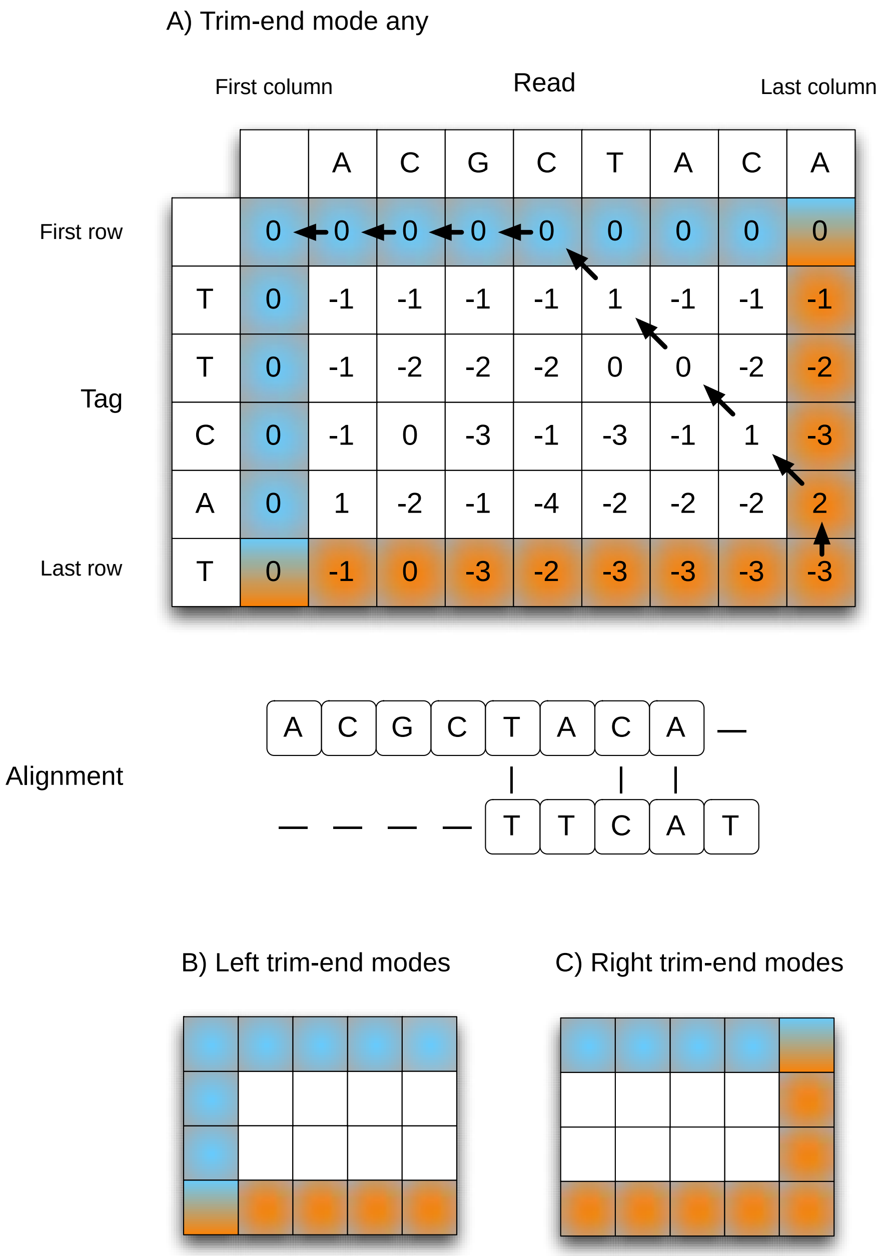

Table S1. Read mapping statistics of benchmark 1—Adapter removal from small RNA-seq data set.

Statistic	FLEXBAR	FASTX	BTRIM	CUTADAPT
Uniquely mappable reads	3162386	3156679	830866	2677830
Non-uniquely mappable reads	1596783	1593109	5005658	1372730
Not mappable reads	4913331	4629118	2637343	7486241

Table S2. Program calls for benchmark 1—Adapter removal from a small RNA-seq data set.

Program	Options
FLEXBAR	-a adapter.fasta -m 20 -r SRR014966.fastq -n 8 -f fastq -t out
FASTX Clipper	-a TCGTATGCCGTCTTCTGCTTGT -l 20 -i SRR014966.fastq -Q33 -o out.fastq
CUTADAPT	-a TCGTATGCCGTCTTCTGCTTGT -m 20 SRR014966.fastq -o out.fastq
BTRIM	-p adapter.fasta -t SRR014966.fastq -o out.fastq -P -S -l 8

Trimmed reads from out.fastq files were mapped back to the *C. elegans* genome with: bowtie -t -m 1 c_elegans_ws200 out.fastq out.map (available at http://bowtie-bio.sourceforge.net).

Table S3. Program calls for benchmark 2—Adapter removal from a 2 × 100 nt RNA-seq data set.

Program	Options
FLEXBAR	flexbar -n 8 -r SRR504324_1.fastq -p SRR504324_2.fastq -t out -f fastq-i1.8 -a PolyAnT.fasta -ae RIGHT -m 20 -x 13 -q 20
FASTX	fastx_trimmer -f 14 -Q33 -i SRR504324_1.fastq | fastq_quality_trimmer -t 20 -Q33 | fastx_clipper -a AAAAAAAAAAAAAAAAAAAAAAAAAAAAAAAAAAAAAAAAAAAAAAAAAAAA -Q33 | fastx_clipper -a TTTTTTTTTTTTTTTTTTTTTTTTTTTTTTTTTTTTTTTTTTTTTTTTTTTT -Q33 -l 20 -o out.fastq

Table S4. Program calls for benchmark 4—Color-space read assignment to splice leaders.

Program	Options
FLEXBAR	flexbar -n 20 -r SRR353599.fasta -t L1_1hr_recovery_ovl10_thr2_rep1 -be LEFT_TAIL -m 18 -bo 10 -b splice_leader_bs.fa --format csfasta -bt 2 -bg -100 > logSRR353599_ovl10_thr2
FLEXBAR decoys	flexbar -n 20 -r SRR353599.fasta -t L1_1hr_recovery_ovl10_thr2_rep1 -be RIGHT_TAIL -m 18 -bo 10 -b splice_leader_bs.fa --format csfasta -bt 2 -bg -100 > logSRR353599_ovl10_thr2

Table S5. Read counts for benchmark 4—Color-space read assignment to splice leaders.

Parameters { -bo , -bt}	SL1	SL1 false	SL2	SL2 false
{20,0}	137020	1729	21030	133
{20,1}	176763	3359	25344	324
{15,1}	181157	10896	26423	1096
{15,2}	201774	17397	33200	2649
{10,1}	228515	22707	34488	5513
{10,2}	276306	48144	60974	52443
